# Isoflurane promotes glucose metabolism through up-regulation of *miR-21* and suppresses mitochondrial oxidative phosphorylation in ovarian cancer cells

**DOI:** 10.1042/BSR20170818

**Published:** 2017-12-12

**Authors:** Nai-Liang Guo, Jia-Xin zhang, Jing-Ping Wu, Ying-Hua Xu

**Affiliations:** 1 Department of Anesthesiology, Zhoupu Hospital, Pudong New District, Shanghai 201318, P.R. China; 2 Department of Anesthesiology, Huadong Hospital Affiliated to Fudan University, Shanghai 200040, China

**Keywords:** Isoflurane, miR-21, mitochondrial oxidative phosphorylation, ovarian cancer cells

## Abstract

Ovarian cancer is one of the most lethal gynecologic malignancies in women. Isoflurane is one of the volatile anesthetics used extensively for inhalational anesthesia and gynecological surgery. However, the effects of isoflurane on ovarian cancer have not been fully elucidated. It is widely studied that one of the biochemical fingerprints of cancer cells is the altered energy metabolism which is characterized by preferential dependence on glycolysis for energy production in an oxygen-independent manner. In the present study, we explored the roles of isoflurane in the regulation of cellular metabolism of ovarian cancer cells. We observed the glucose uptake, lactate production and extracellular acidification of two ovarian cancer cell lines, SKOV3 and TOV21G were significantly stimulated by isoflurane treatments at 1 and 2 h. The glycolysis enzymes, HK2, PKM2, and LDHA were up-regulated by isoflurane. We report that *miR-21* was induced by isoflurane treatments in ovarian cancer cells, leading to the elevated AKT phosphorylation and up-regulation of glycolysis enzymes. In contrast, the mitochondrial functions were suppressed by isoflurane treatments: the oxygen consumption, mitochondrial membrane potential (MMP), and activities of complex I, II, and IV on the electron transport chain were significantly decreased under isoflurane treatments. Importantly, ovarian cancer cells become hypersensitive to glycolysis inhibitors with isoflurane pretreatments. The present study demonstrates that isoflurane treatments drive a metabolic switch of ovarian cancer cells and contributes to the discovery and development of clinical therapeutic agents against ovarian cancer.

## Introduction

Ovarian cancer is one of the most lethal gynecologic malignancies in women [1,2]. The origin and pathogenesis of epithelial ovarian cancer are still poorly understood although they have long been investigated [2]. Currently, the optimal treatment of ovarian cancer is the combination of surgery and chemotherapy [3]. However, metastatic recurrence derived from chemoresistance after treatments is the major cause of mortality.

Isoflurane is one of the volatile anesthetics used extensively in gynecological surgery [4]. Although the exact mechanism of its action has not been clearly delineated, isoflurane has been reported to bind to GABA [5], glutamates [6], and glycine receptors [7]. Moreover, it is known that isoflurane protects the heart against ischemia–reperfusion (I/R) injury [8,9]. Currently, the cellular effects of isoflurane on cancer cell remain elusive. Isoflurane has been described as a stimulator in ovarian cancer cells [10]. Isoflurane exposure significantly increased insulin-like growth factor (IGF)-1 and IGF-1R expression, cell cycle progression, and cell proliferation in SKOV3 cells [10].

Cancer cells exhibit altered cellular metabolism characterized by elevated glycolysis and lactate production but attenuated oxygen consumption in the presence of oxygen, representing one of the ‘hallmarks of cancer’ [11,12]. Compared with normal cells which utilize oxidative phosphorylation as the predominant source of ATP, glycolysis of cancer cells is up-regulated in an adaptive response to oxygen limitation [12]. The metabolic switch not only renders cancer cells growth advance in tumor environments, it also contributes to chemo- or radioresistance through the up-regulation of glycolytic pathway [13]. Therefore, targetting the dysregulated tumor metabolism remains attractive for clinically therapeutic intervention. In the present study, we will explore the roles of isoflurane in the regulation of glycolysis and oxidative phosphorylation of human ovarian cancer cells. The potential mechanisms of how isoflurane may influence ovarian cancer cellular metabolism will be investigated.

## Experimental

### Cell culture and transfection

The human ovarian epithelial carcinoma cell lines SKOV3 and TOV21G were obtained from American Type Culture Collection (ATCC). The human normal ovarian cell line, T1074 was purchased from Abmgood. Cells were cultured in McCoy’s 5A medium (Sigma–Aldrich, Shanghai, China), containing 10% FBS (Thermo Fisher Scientific, Waltham, MA), 2 mM L-glutamine and 1% penicillin (Sigma–Aldrich, Shanghai, China). Cells were cultured in an incubator containing humidified air and 5% CO_2_ at 37°C. The exposure to isoflurane was performed according to a recent description [10]. Briefly, cells were exposed to 2% isoflurane for 1 or 2 h. Under our experimental conditions, 2% isoflurane in gas phase was ~0.42 mM in the aqueous phase when fully equilibrated [10]. The N_2_ exposure was used as a control treatment. Cells were transfected at a final concentration of 50 nM of anti-*miR-21* or control antisense or pre-*miR-21* using Lipofectamine RNAi MAX reagent (Thermo Fisher Scientific, Waltham, MA) according to the manufacturer’s instructions. Seventy-two hours after transfection, cells were collected for the following experiments.

### RNA isolation and quantitative RT-PCR

Total RNA was isolated using TRIzol (Invitrogen, Carlsbad, CA) according to the manufacturer’s protocol. RNA (1 μg) in each treatment was reverse transcribed into cDNA using iScript Reverse Transcription Supermix (Bio–Rad). For detection of miRNAs, the miScript SYBR Green PCR Kit (Qiagen, Shanghai, China) was used for the measurement of *miR-21* expressions. U6 small nuclear RNA was used as an internal control for *miR-21*. Quantitative reverse-transcription PCR (qRT-PCR) was performed with SYBR® Green Quantitative RT-qPCR Kit (Sigma–Aldrich, Shanghai, China) using Bio–Rad CFX96 Real-time PCR detection system. β-actin was used as an internal control for mRNAs (glycolysis enzymes). The primer sequences used in the present study are: GLUT1: forward: 5′-AACTCTTCAGCCAGGGTCCAC-3′; reverse: 5′-CACAGTGAAGATGATGAAGAC-3′; HK2: forward: 5′-CAAAGTGACAGTGGGTGTGG-3′; reverse: 5′-GCCAGGTCCTTCACTGTCTC-3′;

PKM2: forward: 5′-CCACTTGCAATTATTTGAGGAA-3′; reverse: 5′-GTGAGCAGACCTGCCAGACT-3′; LDHA: forward 5′-TTGGTCCAGCGTAACGTGAAC-3′; reverse: 5′-CCAGGATGTGTAGCCTTTGAG-3′; β-actin: forward: 5′-TCCCTGGAGAAGAGCTACGA-3′; reverse: 5′-AGCACTGTGTTGGCGTACAG-3′; U6: 5′-AACGCTTCACGAATTTGCGT-3′. The following conditions were used in the thermal cycle: 50 °C for 30  min, 94 °C for 15  min, followed by 36 cycles at 94 °C for 30  s, 55 °C for 30  s, 72°C for 45  s. The expression of each gene was determined by 2^−ΔΔ*C*^_T_ method using the CFX Manager software.

### Immunofluorescence stating

SKOV3 cells were fixed in cold methanol for 20 min in −20°C freezer followed by treatment with 0.25% Triton-100 at 4°C for 20 min. Cells were blocked with 5% normal BSA solution for 1 h. Samples were then washed three times with PBS and incubated at 4°C overnight with the primary antibody: rabbit anti-GLUT1 (1:200), followed by fluorochrome-conjugated secondary antibodies for 1 h at room temperature. The slides were counterstained with the nuclear dye DAPI and mounted with VECTASHIELD Mounting Medium (Vector Lab, U.S.A.) and immunofluorescence was quantitated using ImageJ (National Institutes of Health, MD, U.S.A.).

### Detection of glycolytic rates

The glucose uptake assay was performed using the Glucose Uptake Assay Kit (Abcam, #ab136955) and lactate production assay was performed using the L-Lactate Assay Kit (Abcam, #ab65331) according to the manufacturer’s protocol. Results were normalized by the protein amounts in each assay. The relative glucose uptake and lactate production of treated group were calculated with the percentage of the control group.

### Measurement of extracellular acidification and oxygen consumption rate

Total cells (4 × 10^4^/well) were plated in XF24 cell culture microplate overnight. Then, the culture medium was replaced with XF assay medium containing 2 mM L-glutamine. Extracellular acidification rate (ECAR) and oxygen consumption rate were measured using XF24 Extracellular Flux Analyzer (Seahorse Bioscience) according to the recent description [14].

### Measurement of intracellular ATP

Cellular ATP contents were measured by using an ATP Assay Kit (Abcam, #ab83355) according to the manufacturer’s protocol. Briefly, 100 μl of the cell lysate was mixed with 100 μl of ATP reaction mix and incubated for 30 min. Absorbance was measured (optical density (OD): 570 nm) by using SpectraMax Microplate Reader (Molecular Devices, Sunnyvale, CA, U.S.A.).

### Measurement of mitochondrial membrane potential and activities of complexes of respiration chain

The mitochondrial membrane potential (MMP) was measured using MitoProbe™ JC-1 Assay Kit (#M34152, Thermo Fisher Scientific, Waltham, MA) as previously described [15]. The activities of complexes on the electron transport chain were measured using Complex I Enzyme Activity Microplate Assay Kit (Abcam, #ab109721); Complex II Enzyme Activity Microplate Assay Kit (Abcam, #ab109908); and Complex IV Human Enzyme Activity Microplate Assay Kit (Abcam, #ab109909) according to the manufacturers’ protocol. Results were normalized by the protein amounts in each assay.

### Cell survival assay

Cell survival assay was measured using the MTT assay. Briefly, confluent SKOV3 cells in 96-well cell culture microplates were treated with different concentrations of glycolysis inhibitors for 48 h at 37°C followed by the addition of 15 μl of MTT solution to each well. The microplate was incubated at 37°C for 4 h. Then, 100 μl of the solubilization/stopping solution was added to each well. The OD of wells was measured at 570 nm using a SpectraMax microplate reader (Molecular Devices, Sunnyvale, CA, U.S.A.).

### Western blotting

Cells were lysed with RIPA buffer containing protease and phosphatase inhibitors cocktail (Roche). After 20-min incubation on ice, the cell lysates were centrifuged at 12000 ***g*** for 15 min at 4°C and the supernatants were collected. The lysates were separated by SDS/PAGE and then transferred on to the nitrocellulose membrane (Bio–Rad). Membranes were blocked with 5% nonfat milk in PBS containing 0.1% Tween-20 (PBS-T) for 1 h at room temperature, the membranes were incubated with antibodies against GLUT1, PKM2, HK2, LDHA (Glycolysis Antibody Sampler Kit #8337, Cell Signaling, Danvers, MA, U.S.A.) and β-actin (#4970, Cell Signaling, Danvers, MA, U.S.A.) at 1:1000 dilution at 4°C overnight. Membranes were washed three times and incubated with secondary antibody (IRDye conjugated IgG, LI-COR) in PBS-T containing 5% nonfat milk for 1 h. The signals were then detected with Odyssey Imaging System (LI-COR).

### Statistics analysis

Statistical analyses were performed using GraphPad Prism 5.0. The statistical significance was determined with a two-tailed Student’s *t* test for unpaired data. *P*-values under 0.05 were considered statistically significant.

## Results

### Isoflurane treatments promote the glycolytic rate in ovarian cancer cells

**Figure 1:**
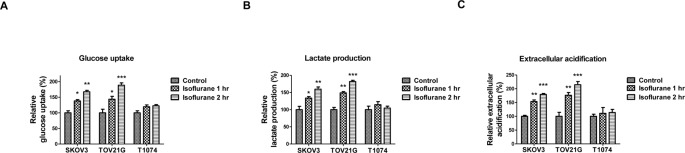
Glycolytic metabolism differences between isoflurane treated and untreated ovarian cancer cells SKOV3, TOV21G, and T1074 cells were treated without or with isoflurane for 1 or 2 h, cells were then cultured in fresh medium for 24 h. The (**A**) glucose uptake, (**B**) lactate production, and (**C**) ECAR were measured in these three groups of cells. All experiments were performed at least three times independently and the data shown are mean ± S.D.; *: *P*<0.05; **: *P*<0.01; ***: *P*<0.001.

Since the metabolic switch from oxidative phosphorylation to glycolysis is a unique characteristic of cancer [11,12], we started to assess the effects of isoflurane treatments on the glucose metabolism in ovarian cancer cells. In ovarian cancer cell lines, SKOV3 and TOV21G, increased glucose uptake, lactate production, and ECAR were observed 24 h after exposure of ovarian cancer cells to isoflurane for 1 or 2 h (Figure 1A–C). Moreover, no significant changes in glucose uptake, lactate production, and ECAR were observed in human normal ovarian cells, T1074 (Figure 1A–C), suggesting isoflurane treatments could stimulate the cellular metabolism in ovarian cancer cells.

### Glycolysis key enzymes were up-regulated by isoflurane treatments

**Figure 2:**
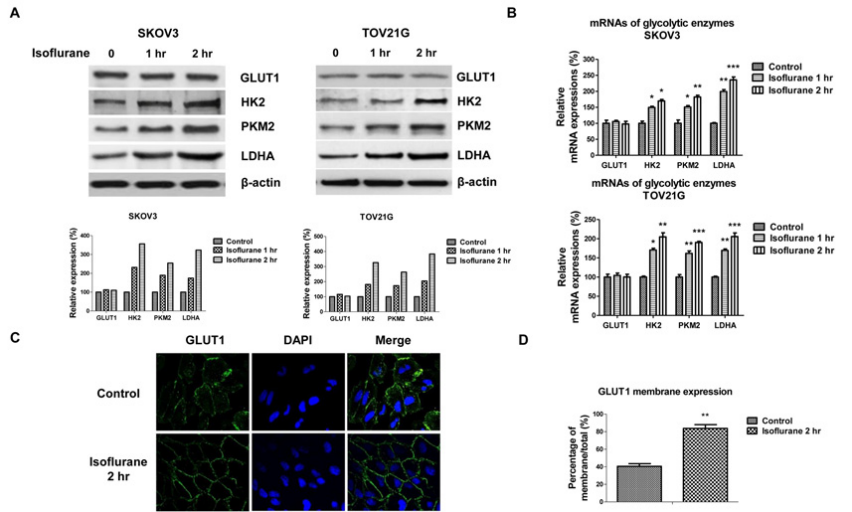
Glycolytic enzymes were activated by isoflurane treatments SKOV3 and TOV21G cells were treated without or with isoflurane for 1 or 2 h, cells were then cultured in new medium for 24 h. (**A**) The protein expressions of GLUT1, HK2, PKM2, and LDHA were measured by Western blot. β-actin was used as a loading control. (**B**) The mRNA expressions of glycolytic enzymes were measured by qRT-PCR. (**C**) SKOV3 cells were treated without or with isoflurane for 2 h, cells were incubated in fresh medium for 24 h. Cells were fixed and stained with antibody against GLUT1. DAPI was used for the nuclei staining. (**D**) Quantitation of the membrane localization of GLUT1 based on the results of Figure 2C using ImageJ software. All experiments were performed at least three times independently and the data shown are mean ± S.D.; *: *P*<0.05; **: *P*<0.01; ***: *P*<0.001.

To assess the metabolic pathway which is up-regulated by isoflurane treatments, we measured the glucose metabolic enzymes expression in SKOV3 and TOV21G cells by Western blot or qRT-PCR. As we expected, isoflurane treatments significantly up-regulated both protein and mRNA expressions of HK2 and PKM2, whose overexpression in cancer cells resulted in up-regulated aerobic glycolysis [11] and LDHA, which catalyzes the interconversion of pyruvate and L-lactate [11] (Figure 2A,B). The expressions of GLUT1, which is a glucose transporter localizing on the plasma membrane did not change (Figure 2A,B). However, isoflurane treatments significantly increased the translocation of GLUT1 to membrane (Figure 2C,D). Taken together, these data demonstrated isoflurane treatments promote glycolytic rate in ovarian cancer cells.

### Isoflurane up-regulates glycolysis enzymes expression through *miR-21*-AKT pathway

**Figure 3:**
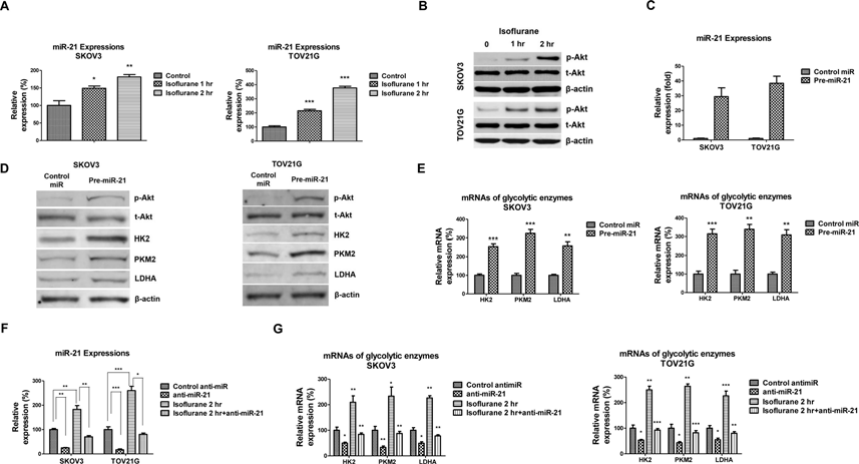
Isoflurane induces *miR-21* expression and phosphorylation of AKT (**A**) SKOV3 and TOV21G cells were treated without or with isoflurane for 1 or 2 h, and the expressions of *miR-21* were assessed by qRT-PCR. U6 was used as an internal control. (**B**) SKOV3 and TOV21G cells were treated with isoflurane for 1 or 2 h, the phosphorylation of AKT was measured by Western blot. β-actin was used as the loading control. (**C**) SKOV3 and TOV21G cells were transfected with control miRNAs or pre-*miR-21* for 72 h. The expression of *miR-21* was assessed by qRT-PCR. U6 was used as the internal control. (**D**) The proteins and (**E**) mRNAs of HK2, PKM2, and LDHA were measured in SKOV3 and TOV21G cells without or with *miR-21* overexpression. (**F**) SKOV3 and TOV21G cells without or with isoflurane treatment were transfected with control antisense or anti-*miR-21* for 72 h. The expression of *miR-21* was assessed by qRT-PCR. U6 was used as an internal control. (**G**) The mRNAs of GLUT1, HK2, PKM2, and LDHA were measured by qRT-PCR. All experiments were performed at least three times independently and the data shown are mean ± S.D.; *: *P*<0.05; **: *P*<0.01.

To investigate the mechanisms for the isoflurane up-regulated glucose metabolism enzymes, we measured the expressions of *miR-21* in SKOV3 and TOV21G cells under isoflurane treatments since recent publications described that isoflurane could induce the *miR-21* expression in cardiomyocytes [9,15]. As we expected, *miR-21* is significantly up-regulated by isoflurane treatments (Figure 3A), suggesting that *miR-21* might involve in isoflurane-regulated glycolysis in ovarian cancer cells. We next measured the AKT pathway which has been reported to positively regulate glycolysis [16]. Results in Figure 3B illustrated that isoflurane could induce the phosphorylation of AKT. We transfected *miR-21* into ovarian cancer cells (Figure 3C) and found that overexpression of *miR-21* significantly up-regulated Akt phosphorylation (Figure 3D). The glycolysis enzymes HK2, PKM2, and LDHA were significantly up-regulated at protein levels and mRNA levels by *miR-21* overexpression (Figure 3D,E). Moreover, inhibition of *miR-21* suppressed both basal level and isoflurane induced glycolysis enzymes expressions (Figure 3F,G), indicating that isoflurane-promoted glycolytic rate was through up-regulation of *miR-21*. Taken together, the above results demonstrated an isoflurane–*miR-21*–AKT–glycolysis axis in ovarian cancer cells.

### Mitochondrial functions of ovarian cancer cells are suppressed by isoflurane treatments

**Figure 4:**
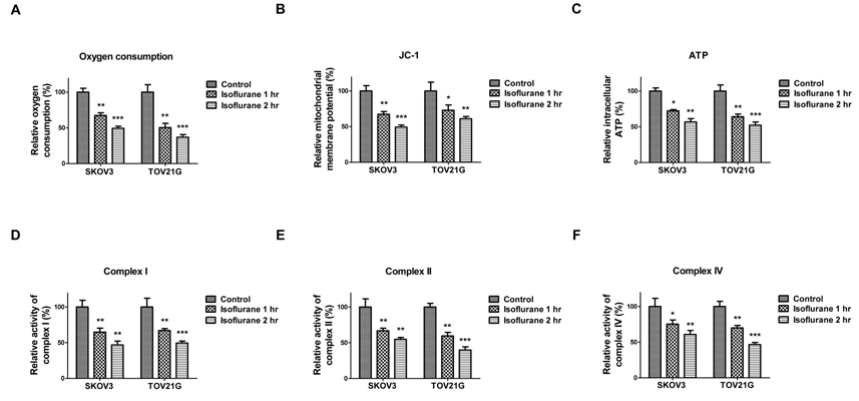
Isoflurane suppresses mitochondrial functions (**A**) SKOV3 and TOV21G cells were treated without or with isoflurane for 1 or 2 h, cells were then cultured in new medium for 24 h. The (A) oxygen consumption; (**B**) MMP; (**C**) intracellular ATP; (**D**) activity of complex I on the electron transport chain; (**E**) activity of complex II on the electron transport chain, and (**F**) activity of complex IV on the electron transport chain were compared. All experiments were performed at least three times independently and the data shown are mean ± S.D.; *: *P*<0.05; **: *P*<0.01; ***: *P*<0.001.

As we discussed above, rather than oxidative phosphorylation in mitochondria, predominant glycolysis is a common metabolic property in cancer cells. Our results in Figures 1 and 2 demonstrated ovarian cancer cells under isoflurane exposure display elevated glycolysis, leading us to hypothesize that isoflurane might make a metabolic switch of ovarian cancer cells through suppression of mitochondrial oxidative phosphorylation. To test this, we compared oxygen consumption rates, which reflect the globule mitochondrial respiration between control and isoflurane-treated ovarian cancer cells. Oxygen consumption rates were suppressed by isoflurane exposure of 1 or 2 h (Figure 4A). Moreover, the MMP was decreased in isoflurane treated cells. JC-1 assay showed that isoflurane reduced levels of MMP in SKOV3 and TOV21G cells (Figure 4B). We observed at global level, despite enhanced glycolysis, ATP levels in SKOV3 and TOV21G cells were declined following a 1 or 2 h isoflurane treatments (Figure 4C), suggesting that the isoflurane-treated cancer cells might attempt to overcome the mitochondrial ATP shortage by up-regulating glycolysis but could not obtain full recovery of the ATP deficiency. Consistently, the activities of complexes I, II, and IV from mitochondrial respiration chain were decreased by isoflurane treatments (Figure 4D-F). The above data demonstrated that isoflurane could inhibit mitochondrial functions in ovarian cancer cells.

### Ovarian cancer cells are more sensitive to glycolysis inhibition under isoflurane treatments

**Figure 5:**
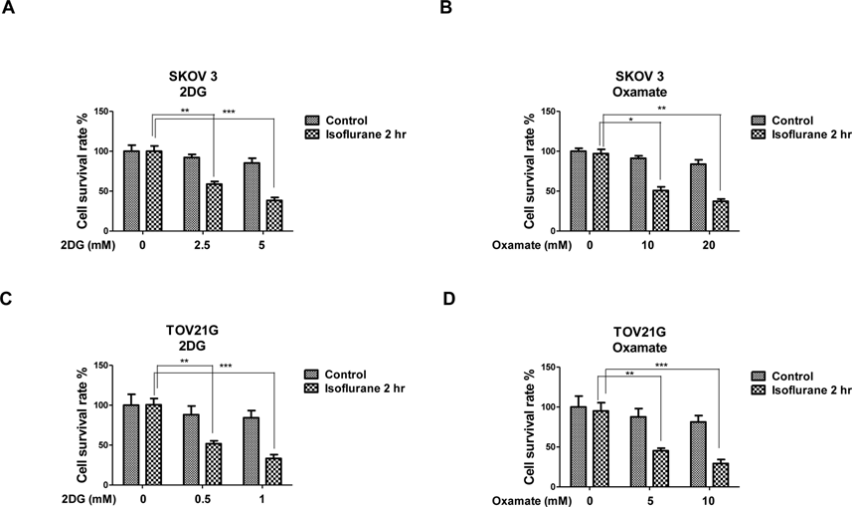
Ovarian cancer cells were more sensitive to glycolysis inhibitors with isoflurane treatments SKOV3 and TOV21G cells were treated without or with isoflurane for 1 or 2 h, cells were then cultured in new medium for 24 h. SKOV3 cells were treated with (**A**) 2DG at 0, 2.5, or 5 mM for 48 h or (**B**) oxamate at 0, 10, or 20 mM for 48 h, followed by the measurements of cell survival by MTT assay. TOV21G cells were treated with (**C**) 2DG at 0, 0.5, or 1 mM for 48 h or (B) oxamate at 0, 5, or 10 mM for 48 h, followed by the measurements of cell survival by MTT assay. All experiments were performed at least three times independently and the data shown are mean ± S.D.; *: *P*<0.05; **: *P*<0.01; ***: *P*<0.001.

Our observations collectively demonstrated that the isoflurane treated cancer cells may rely more on glycolysis for supplementary energy production. Therefore, they might be hypersensitive to glycolysis inhibition. To test this hypothesis, we treated SKOV3 and TOV21G cells with 2DG or oxamate, both are glycolysis inhibitors targetting the hexokinase that catalyzes the first step of glycolysis or LDHA that converts the glycolytic end product pyruvate into lactate and provides NAD^+^ to glycolysis [17]. We found ovarian cancer cells were more sensitive to 2DG or oxamate under isoflurane treatments (Figure 5A–D). At physiological dosages, which did not dramatically induce cell death, SKOV3 and TOV21G cells became sensitive to 2DG or oxamate under isoflurane exposure (Figure 5A–D). Together these results support our hypothesis that isoflurane treatments switch cancer cells metabolism from oxidative phosphorylation to glycolysis for viability and proliferation.

## Discussion

In the present study, we investigated the roles of isoflurane in the regulation of ovarian cancer cells metabolism. Currently, limited laboratory studies on the direct effects of specific anesthetic agents such as isoflurane on cancer cell biology have been described. It has been reported that serum of breast cancer surgery patients who received propofol-paravertebral anesthesia inhibited proliferation of one type of breast cancer cells *in vitro* [18]. In addition, a recent study demonstrated ovarian cancer cells exposed to isoflurane displayed up-regulated cell cycle progression, angiogenesis, and cell proliferation *in vitro* [10], suggesting isoflurane increases the malignant potential in ovarian cancer cells. Our results, consistent with the recent study [10], demonstrated a metabolic switch in ovarian cancer cells under isoflurane treatments, contributing to the malignancy of cancer cells.

Previous studies described that *miR-21* promoted proliferation, invasion, and migration of ovarian cancer cells through inhibiting the expression of PTEN protein [19]. In addition, another group reported targetting *miR-21-3p* inhibits proliferation and invasion of ovarian cancer cells [20], suggesting that inhibiting *miR-21* contributes to antiovarian cancer therapy. Consistently, our study demonstrated that overexpression of *miR-21* up-regulated Akt pathway, resulting in dysregulated cellular metabolism. These discoveries revealed that *miR-21* could be a biomarker for prognosis and diagnosis of ovarian cancer and targetting *miR-21* for cancer treatments is likely to improve the outcomes of patients.

Glucose metabolism in cancer cells is primarily characterized by two major biochemical events: increased glucose uptake and lactate production [11,12], which has been recognized as one of the ‘hallmarks of cancer’. Moreover, oxidative phosphorylation is essential for all the cells [21]. We observed isoflurane treatments up-regulated glucose uptake and lactate production. Meanwhile, the mitochondrial functions were suppressed by isoflurane. Importantly, our data showed increased sensitivity of ovarian cancer cells to glycolysis inhibitors under isoflurane treatments, suggesting a potential role of isoflurane in the treatments of clinical ovarian cancer patients. Because the clinical utility of 2DG or oxamate for treatment of cancer requires demonstration of safety, and absence of toxicity at clinically effective doses, our results demonstrated that under isoflurane exposure, relatively lower concentrations of glycolysis inhibitors are required to inhibit cancer cells *in vitro*, providing a new perspective for the clinical utilization of glycolysis inhibitors. However, the mechanisms for this phenomenon are still under investigation.

It has been reported that isoflurane treatments could induce opening of mitochondrial permeability transition pore (mPTP), increase in levels of reactive oxygen species and reduction in levels of MMP in mice brain [22], suggesting a putative function of isoflurane in human ovarian cancer cells. Since the MMP, cellular ATP, and activities of complexes I, II, and IV of the electron transport chain were decreased, it is possible that isoflurane sensitizes cancer cells to glycolysis inhibitors through suppression of energy supply. The direct cellular mechanisms of anesthetics on cancer cell biology remain elusive and detailed *in vivo* research will be performed in our next project. Although we illustrated the effects of isoflurane on *miR-21* and cellular metabolism in multiple ovarian cancer cells, these *in vitro* studies did not actually reflect the microenvironments of human ovarian tumor. Moreover, xenograft mouse models derived from human ovarian cancer cells are performed in immunodeficient mouse strains, the immune response is completely absent from xenograft tumors, presenting a major limitation using xenograft mouse model. In general. these *in vitro* and xenograft mouse model have drawbacks such as difference in biokinetics parameters or extrapolation of results to human, limiting our study at the stage of transiting animal experiments to clinical application.

In summary, we explored the roles of isoflurane in the cellular metabolism of ovarian cancer cells and underlined mechanisms of how isoflurane regulates cellular metabolism. The present study first provides supports for illustrating isoflurane treatments could drive a metabolic switch and contributes to the discovery and development of clinical therapeutic agents against ovarian cancer.

## Abbreviations

AKT: protein kinase B
ECAR: extracellular acidification rate
GABA: γaminobutyric acid
GLUT1: Glucose transporter 1
IGF-1R: insulin-like growth factor 1 receptor
MMP: mitochondrial membrane potential
OD: optical density
PBS-T: PBS containing 0.1% Tween-20
PTEN: phosphatase and tensin homolog
qRT-PCR: quantitative reverse-transcription PCR
RIPA: Radio-Immunoprecipitation Assay
2DG: 2-deoxy-D-glucose
